# A novel comparison of erector spinae plane block and paravertebral block in laparoscopic cholecystectomy

**DOI:** 10.1590/1806-9282.20231457

**Published:** 2024-04-22

**Authors:** Elvan Tekir Yılmaz, Duygu Demiriz Gülmez, Alparslan Apan, Bilge Olgun Keles, Mücahit Coşkun, Cihan Döger, Tugrul Kesicioglu, Vedat Ataman Serim, Furkan Ali Uygur, Ilker Sengul

**Affiliations:** 1 Giresun University, Faculty of Medicine, Department of Anesthesiology and Reanimation – Giresun, Turkey.; 2 Başakşehir Çam and Sakura City Hospital – İstanbul, Turkey.; 3 University of Health Sciences, Faculty of Medicine, Department of Anesthesiology and Reanimation – Ankara, Turkey.; 4 Giresun University, Faculty of Medicine, Department of General Surgery – Giresun, Turkey.; 5 Giresun University, Faculty of Medicine, Department of Neurology – Giresun, Turkey.; 6 Giresun University, Faculty of Medicine, Division of Endocrine Surgery – Giresun, Turkey.

**Keywords:** Paravertebral, Block, Laparoscopic cholecystectomy, Postoperative pain

## Abstract

**OBJECTIVE:**

Erector spinae plane block is an updated method than paravertebral block, possessing a lower risk of complications. This study aimed to compare erector spinae plane and paravertebral blocks to safely reach the most efficacious analgesia procedure in laparoscopic cholecystectomy cases.

**METHODS:**

The study included 90 cases, aged 18–70 years, classified as American Society of Anesthesiologists I–II, who underwent an laparoscopic cholecystectomy procedure. They were randomly separated into three groups, namely, Control, erector spinae plane, and paravertebral block. No block procedure was applied to Control, and a patient-controlled analgesia device was prepared containing tramadol at a 10 mg bolus dose and a 10-min locked period. The pain scores were recorded with a visual analog scale for 24 h postoperatively.

**RESULTS:**

The visual analog scale values at 1, 5, 10, 20, and 60 min at rest and 60 min coughing were found to be significantly higher in Control than in paravertebral block. A significant difference was revealed between Control vs. paravertebral block and paravertebral block vs. erector spinae plane in terms of total tramadol consumption (p=0.006). Total tramadol consumption in the first postoperative 24 h was significantly reduced in the paravertebral block compared with the Control and erector spinae plane groups.

**CONCLUSION:**

Sonography-guided-paravertebral block provides sufficient postoperative analgesia in laparoscopic cholecystectomy surgery. Erector spinae plane seems to attenuate total tramadol consumption.

## INTRODUCTION

Laparoscopic procedures have opted for conventional ones for reasons such as causing less surgical trauma, pain, wound site infection, and fewer postoperative respiratory complications, providing better cosmetic results, and allowing early mobilization and discharge from the hospital^1-3^. However, pain remains a challenge for laparoscopic cholecystectomy (LC) cases, although postoperative pain rates are lower than in conventional ones. Given this, opioids and non-steroid anti-inflammatory drugs are frequently selected for the elimination of pain, but they may cause dose-dependent side effects such as nausea, itching, kidney failure, respiratory depression, and addiction, or the analgesic efficacy may remain insufficient. In addition, an increase in the use of interfascial blocks and nerve blocks in postoperative pain has been engaged together with the spread of ultrasonography utilization^4^. Paravertebral block (PVB), per se, has been widely used in postoperative analgesia for many years and has been shown to improve pain scores, attenuate the essentiality for additional analgesia, and improve respiratory function^5-7^. However, the proximity of PVB to the pleura limited its usage due to potential complications, but with the augmented use of sonography, this complication risk has diminished^8^. Although there is some controversy about the effect mechanism of erector spinae plane (ESP) block, its efficacy has been proven by many studies^9,10^. To the best of our knowledge, no study has yet been published that has compared the efficacy and complications of ESP block and PVB in LC operations. The primary aim of this study was to compare ESP and PVB blocks as important postoperative pain management in terms of being able to reliably reach the highest analgesic efficacy in patients who underwent LC, which is a frequently applied surgery.

## METHODS

### Ethical aspects

This study was approved by the Ethics Committee of Clinical Researchers linked to Ordu University, under approval number 0685021.283/2021.

### Study design

**Figure 1:**
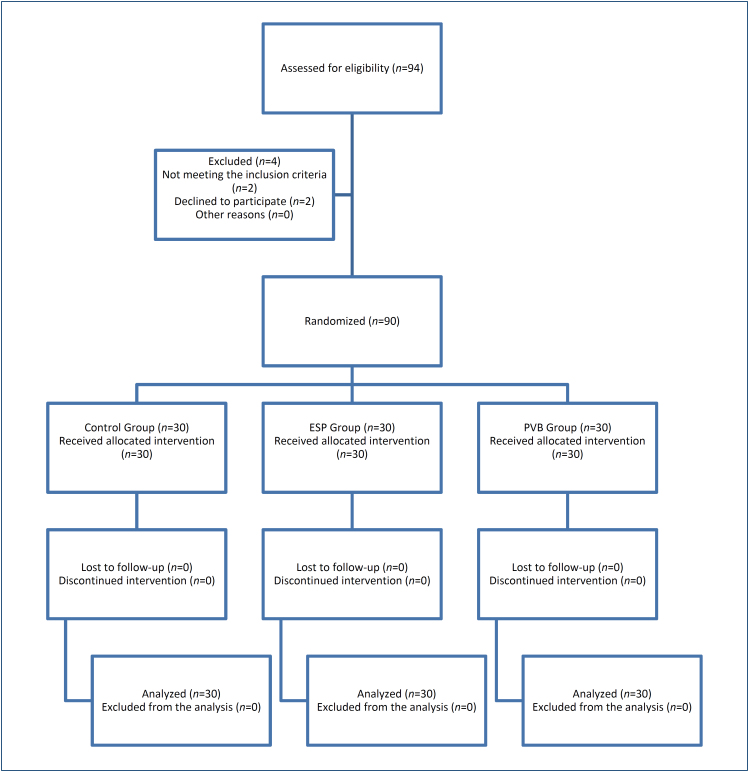
Consolidated standards of reporting trials flow diagram.

This prospective randomized, controlled study was conducted between January 2022 and October 2022 using a consolidated standards of reporting trials (CONSORT) flow diagram shown in Figure 1 for the recording of patients (Figure 1). The study incorporated a total of voluntary basis as those aged >18 years of physical status American Society of Anesthesiologists (ASA) I–II, who were planned to undergo LC. The exclusion criteria were as follows: (i) not providing informed consent, (ii) possessing any psychiatric or mental problem that prevented understanding of the informed consent form, (iii) planning to undergo an emergency cholecystectomy procedure, (iv) having any allergy or hypersensitivity to local anesthetic (LA), (v) possessing an infection in the needle entry area, and (vi) having a history of coagulopathy or the use of anticoagulants. All patients underwent routine general anesthesia protocol in our clinic. Patients were divided into three groups, namely, Control, ESP, and PVB.

### Erector spinae plane block technique

**Figure 2:**
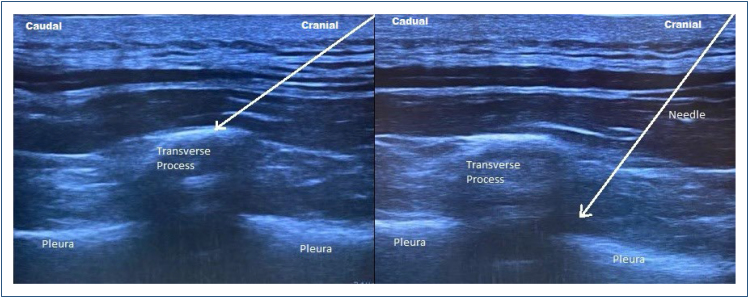
**(A)** A sonographic imaging of thoracal paravertebral block (the arrow indicates the location of the needle, placed craniocaudally within the fascial plane of the deep surface of the erector spinae muscle above the bone shadow of the transverse process). **(B)** A sonographic imaging of erector spinae block (the arrow indicates the location of the needle, placed in the paravertebral space above the pleura). PVP: paravertebral block; ESP: erector spinae block.

After sterilization of the skin with povidone-iodine, the probe covered with a sterile sheath was placed 3 cm lateral of the T8 spinous process. The trapezius, rhomboid major, and erector spinae muscles and the transverse process (TP) of the vertebrae were visualized, and the needle was placed craniocaudally within the fascial plane of the deep surface of the erector spinae muscle above the bone shadow of the TP. The fluid dissemination was confirmed by raising the placement of the needle tip toward the erector spinae muscle and a 20 mL of 0.25% bupivacaine was applied to this region and the spread of LA was observed (Figure 2A).

### Paravertebral block technique

The spinous processes of the vertebrae had been marked up to the T8 level. After providing an antisepsis of the skin with 10% povidone-iodine, the ultrasound probe was placed parallel to the vertebral spine at T8. The TP and hyperechoic pleura were observed 2.5 cm right lateral of the spinous process, and the needle was placed in the caudal direction by using the in-plane approach. Furthermore, 20 mL of 0.25% bupivacaine was administered for the block after confirming displacement of the pleura with 0.5–1 mL LA (Figure 2B).

### Postoperative pain management

All the cases had been followed up for 30 min in the Postanesthetic Care Unit. Patients with a visual analog scale (VAS) score of ≥4 had been administered 50 mg tramadol. Of note, a patient-controlled analgesia (PCA) device (BodyGuard 595 ColorVision Pump/Belgium) was prepared with tramadol. The infusion dose was not adjusted and was set as 10 mg bolus with a 10-min locked period. In the follow-up of the patients on the ward, 1 g paracetamol had been administered as routine at the sixth hour. A 50-mg dexketoprofen trometamol (Arveles^â^, 50 mg/2 mL, Menarini Co., Ltd., Istanbul, Turkey) was administrated as rescue analgesia during follow-up, whereas 1 mg morphine (MorfinHidroklorür^â^, 0.01 g/1 mL Osel Co., Ltd., Istanbul, Turkey) was performed if VAS score did not reduce to <4 within half an hour. At postoperative 1, 4, 12, 18, and 24 h, the VAS scores and tramadol bolus dose were recorded. All the patients were questioned about nausea, vomiting, and shoulder pain.

### Sample size determination

The sample size was determined because of power analysis performed using the G*Power 3.1.9.7 software^11^. For the analysis of variance (ANOVA) test, it was determined that at least 28 cases were required in each group to give a moderate effect size (f=0.35) as calculated in previous studies^9,12,13^ with type 1 error (alpha) of 0.05 and type 2 error (beta) of 0.80. Taking possible losses of 10% into consideration, a sum of 90 patients were included in the study, with 30 in each of the three groups.

### Statistical analysis

Data obtained in the study were statistically analyzed using the Statistical Package for Social Sciences (SPSS)25 software (SPSS Inc., Chicago, IL, USA). The conformity of the continuous variables to normal distribution was assessed with the Shapiro-Wilk test, and the subsequent data were stated as mean±standard deviation (SD) values and categorical data as number (n) and percentage (%). The Pearson chi-square test was used to compare categorical data between groups, and the ANOVA test was applied in comparisons of data showing normal distribution. The post hoc Tukey test was applied to determine from which group the difference originated for the parameters where a significant difference was recognized. The subsequent data not exhibiting the normal distribution were compared with the Kruskal-Wallis test. Moreover, the Mann-Whitney U test was applied in paired comparisons to determine the group that was the source of the parameters where a statistically significant difference emerged. Finally, a value of two-tailed p<0.05 was accepted as statistically significant in all the statistical comparisons.

## RESULTS

A posteriori, an evaluation was made of a total of 90 cases, comprising 56 (62.2%) females and 34 (37.8%) males with a mean age of 53.2±12.7 years. Herein, all groups were similar with respect to gender distribution, BMI, ASA, duration of anesthesia, surgery, and duration of block application. The HR values at 5, 10, and 20 min after extubation were found to be higher in Control than in ESP (p<0.017). The other hemodynamic parameters were similar in all. The VAS values at 0, 5, 10, 20, and 60 min at rest and at 60 min coughing were found to be significantly higher in Control than in PVB. There is a statistically significant difference in VAS values of only 20 min between the Control and ESP groups. At the other time points, no statistically significant difference between the VAS values of the groups had been recognized. The data of postoperative nausea, gas output, mobilization, shoulder pain, time of first food intake, and time to discharge were similar in the groups. The preference status and satisfaction of the patients in Control were found to be lower than those of the patients in the block groups. The mean tramadol consumption throughout the first 24 h postoperatively was 84.7±98.5 mg in Control, while it was 57.7±83.7 mg in ESP and 21.7±48.6 mg in PVB. A statistically significant difference was determined between Control and PVB or between ESP and PVB. Nevertheless, no statistically significant difference was determined between the ESP and Control groups (p>0.05). No patient required dexketoprofen trometamol or morphine during or after recovery. In the comparisons of the postoperative tramadol consumption of the groups, no significant difference was determined with respect to the first 30-min, 4-h, and 24-h consumptions. The tramadol consumption of Control cases was greater compared with PVB at the first, second, and sixth hours, and PVB was lower than that of the other two at the 12th hour. A sum of 12 patients in Control needed it, which was consistent with the VAS scores, in the first hour (Table 1).

**Table 1:** Comparisons of the groups with respect to the postoperative requirement for tramadol.

Time (postoperative)	Control n=30	ESP n=30	PVB n=30	p
30th min	6 (20.0%)	5 (16.7%)	2 (6.7%)	0.311
1st hour	12 (40.0%)^a^	5 (16.7%)	2 (6.7%)	**0.005**
2nd hour	12 (40.0%)^a^	7 (23.3%)	2 (6.7%)	**0.009**
4th hour	8 (26.7%)	7 (23.3%)	4 (13.3%)	0.420
6th hour	14 (46.7%)^a^	7 (23.3%)	4 (13.3%)	**0.013**
12th hour	8 (26.7%)	5 (16.7%)	0 (0.0%)^a^ ^b^	**0.012**
24th hour	4 (13.3%)	2 (6.7%)	1 (3.3%)	0.338
Total	18 (60.0%)	15 (50.0%)	7 (23.3%)^a^ ^b^	0.013

Values are given as number and percentage of cases. The chi-square test was used in the comparisons. A value of p<0.05 was accepted as statistically significant and is mentioned in bold.

a Difference between the Control and PVB groups p<0.05.

b Difference between the PVB and ESP groups p<0.05. PVB: paravertebral block; ESP: erector spinae plane block.

## DISCUSSION

This study was aimed to compare postoperative analgesia requirements, side effects, and complication rates in cases undergoing LC surgery with PVB, which has proven efficacy, and ESP block, the efficacy of which has been attempted to be shown in various studies. This study revealed that the total tramadol consumption in the postoperative 24 h was significantly diminished in the PVB compared with the Control and ESP groups. It was determined that less tramadol was consumed by the PVB than by the other groups at 12 h. Tramadol consumption of the PVB was 0 at the 12th hour. Some authors reported that PVB was applied preoperatively and postoperatively in LC surgery and compared with Control, and the VAS scores and requirement for additional analgesia in both were significantly low compared with Control^12^. This study supports previous studies that have shown PVB to be a very good option for postoperative analgesia^14,15^. This study revealed significant differences between PVB and ESP, or between PVB and Control with respect to the 24-h total tramadol consumption (p<0.006), while the tramadol consumption of Control was greater compared with PVB at the first, second, and sixth hour. PVB has been shown to provide more effective analgesia because of the anatomic proximity to the sympathetic chain that the ESP block did not provide as effective analgesia as PVB, which in this study can be attributed to the surgical areas. Application of a bilateral ESP block might provide better outcomes as abdominal operations such as LC might lead to more widespread pain. Some authors reported that the first 30- and 60-min VAS scores were found to be high when PVB was applied to LC cases, which was attributed to not using any non-opioid agent other than paracetamol^16^. In this study, the VAS scores of the cases were recorded four times in the first 20 min, and the VAS values at 0, 5, 10, 20, and 60 min at rest and at 60 min coughing were higher in Control than PVB. There was no statistically significant difference between the Control and ESP groups. The number of patients in Control requiring analgesia in the first hour was greater and the VAS scores were higher, whereas, at the other time points, the significant difference disappeared and the number of patients requiring additional analgesia decreased which probably occurred secondary to the increased opioid consumption of Control not applied with block and no complications have been developed during or after the procedure. In this study, to better determine the risk–benefit ratio, we purposed to obtain a better result by adding a satisfaction rating that globally evaluates performance (mobilization time) and pain^14^. Patient preference and satisfaction were significantly lower in the control group. We postulate that the lower level of satisfaction in the non-block group in terms of preference and satisfaction makes the results more meaningful.

### Limitations

This study has some limitations. As the block applications had been performed without awakening the patients in the postoperative period, sensory tests could not be performed to find dermatomal spread.

## CONCLUSION

To the best of our knowledge, this is the first study on the efficacy and complications of ESP and PVB in LC cases. The outcomes of this study indicate that 24-h tramadol consumption is lower in PVB than Control and ESP with a significant superiority in PVB compared with Control and ESP. We postulate that the so-called unilateral ESP block concept might not provide adequate postoperative analgesia in LC surgery. We might point out that the complication rate is lower when PVB is performed under sonographic guidance and PVB can be used for postoperative analgesia in LC surgery.
